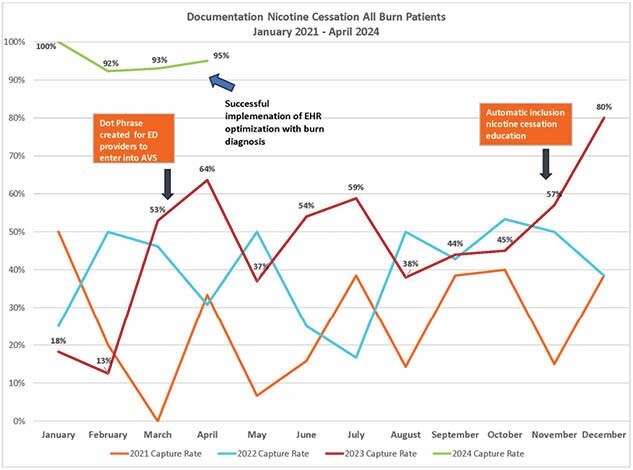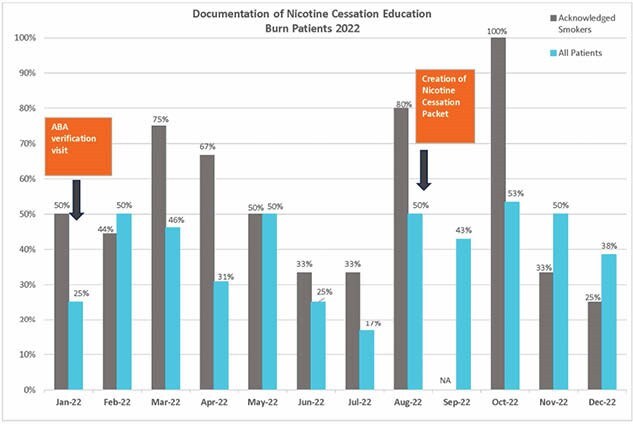# 957 Nicotine Cessation Discharge Documentation for Adult Burn Patients in the ED

**DOI:** 10.1093/jbcr/iraf019.488

**Published:** 2025-04-01

**Authors:** Pamela Michelli, Howard Smith, Susan Smith, Krista Card

**Affiliations:** Orlando Health Regional Medical Center; Orlando Health Regional Medical Center; Orlando Health Regional Medical Center; Orlando Health Regional Medical Center

## Abstract

**Introduction:**

Nicotine use in burn patients leads to higher rates of wound infections, delayed healing, and increased readmissions. Studies show that ED patients have a higher incidence of nicotine use, making cessation education in the ED effective. Historically, the ED provided tobacco cessation only to burn patients who reported tobacco use. In 2022, an opportunity was recognized to formalize written nicotine cessation protocols to include all burned patients discharging directly from the ED. The purpose of the project was to optimize the electronic health record (EHR) to include nicotine cessation in the ED discharge instructions to be distributed to all burn patients discharged from the ED. The hypothesis: EHR changes have improved smoking cessation education for adult burn patients discharged from the ED.

**Methods:**

A literature review on adult burn patients exposed to tobacco was completed in August 2021. Nicotine cessation education, initially tobacco cessation, was developed for all burn patients discharged from the ED. A discharge education packet was created and approved by the multidisciplinary team but was unable to be uploaded due to EHR limitations. A dot phrase was created for ED providers March 2023 to include in discharge instructions, but documentation remained inconsistent. In November 2023, the EHR team enhanced documentation by implementing a standardized nicotine cessation phrase automatically included in discharge paperwork for patients with specific burn diagnosis codes and removed previous barriers. To evaluate the efficacy of nicotine cessation education, medical records for all adult burn discharges from the ED in a single verified Burn Center were reviewed from January 1, 2021, to May 1, 2023.

**Results:**

A review of medical records from January 1, 2021, to May 1, 2023, identified 550 adult burn patients discharged from the ED. In 2021, documentation of tobacco cessation for smokers averaged 47%, and for all patients, 26%. In 2022, new protocols raised these rates to 54% for smokers and 40% for all patients. After optimizing nicotine cessation documentation in the EHR November 2023, compliance improved to 92%.

**Conclusions:**

Nicotine cessation education is imperative for burn healing and long-term health. Finding ways to consistently disseminate and document nicotine cessation in a small patient population such as burn patients requires diligent monitoring and adaptation. EHR optimization improved the nicotine cessation education documentation to an average rate of 92%.

**Applicability of Research to Practice:**

To enhance nicotine cessation education and patient outcomes, optimization of the EHR will prompt providers to document this education. Data will be reviewed to track compliance and establish a feedback loop for continuous improvement. Data will be used to follow up on changes in smoking behavior within the burn population. These strategies will significantly improve the effectiveness of nicotine cessation education for burn patients.

**Funding for the Study:**

N/A